# Multi-drug resistant *Mycobacterium tuberculosis* in Port Harcourt, Nigeria

**DOI:** 10.4102/ajlm.v7i2.805

**Published:** 2018-12-06

**Authors:** Kome Otokunefor, Tosanwumi V. Otokunefor, Godwin Omakwele

**Affiliations:** 1 Department of Microbiology, Faculty of Science, University of Port Harcourt, Port Harcourt, Rivers State, Nigeria

## Abstract

**Background:**

In past years, much focus has been on tackling the scourge and spread of tuberculosis worldwide. The recent emergence of multi-drug resistant (MDR) tuberculosis has, however, negatively threatened progress made so far. Nigeria ranks fourth out of the 22 high tuberculosis burden countries in the world and has the highest burden of tuberculosis in Africa. It is therefore necessary to monitor the MDR tuberculosis situation in the country.

**Objectives:**

This study set out to assess the proportions of MDR tuberculosis in patients attending six directly observed treatment short-course centres in Port Harcourt, Nigeria, from October 2015 to October 2016.

**Methods:**

Six hundred and nine participants between the ages of 18 and 75 years were enrolled in this study and comprised suspected and newly diagnosed tuberculosis cases. Sputum samples obtained from the participants were screened for the presence of *Mycobacterium tuberculosis* using standard culture and phenotypic biochemical techniques, and drug susceptibility testing was carried out using the 1% proportion conventional method.

**Results:**

Of the 609 participants enrolled, 30 (4.9%) were confirmed as *M. tuberculosis*-positive cases. A high prevalence of drug resistant tuberculosis was noted in this study (14/30, 46.7%), with 26.7% of isolates resistant to streptomycin. MDR tuberculosis, defined as being resistant to isoniazid and rifampicin, was detected in only one case (3.3%).

**Conclusion:**

This study reports a low rate of MDR tuberculosis and contributes to the sparse data on drug resistant tuberculosis in Nigeria.

## Introduction

Tuberculosis, caused by *Mycobacterium tuberculosis*, is still a major public health issue worldwide. This disease is particularly problematic in low and middle-income countries, where it is a major contributor to morbidity and mortality.^1^ Of the 1 million people killed by tuberculosis each year, most are from these low and middle-income areas, with sub-Saharan Africa known to have the highest tuberculosis incidence.^2^ The problem of tuberculosis has been compounded in recent years by the emergence of multi-drug resistant (MDR) strains. This phenomenon has been increasingly observed round the globe. In 2015, the World Health Organization (WHO) noted about 480 000 cases of MDR tuberculosis globally,^3^ which accounts for about 5% of all global tuberculosis cases.^4^ The issue of MDR tuberculosis is particularly worrisome, because of its possible negative effect on treatment outcomes (such as treatment failures) and treatment options, with fewer drugs effective against the disease. The current WHO guidelines for the treatment of MDR tuberculosis include the use of at least five different drugs and a treatment time of up to 24 months.^5^ Despite this, a high mortality rate is associated with MDR tuberculosis. One key step in the prevention of drug resistance is continuous monitoring of the situation through systematic surveillance and drug resistance testing.

In a WHO 2016 publication, Nigeria was shown to rank fourth out of the 22 high tuberculosis burden countries worldwide and has the highest burden of tuberculosis in Africa. It is also one of 10 countries that account for 77% of the difference between WHO estimation and actual notifications due to underreporting and underdiagnosis.^3^ Therefore, it is essential to monitor the MDR tuberculosis situation in the country. In the absence of the roll-out of nationwide tuberculosis data, current information is usually obtained from regional studies. Two 2017 reviews analysing drug resistance and multi-drug resistance in Nigeria and sub-Saharan Africa highlighted a gap in knowledge.^6,7^ Of the papers included in these reviews, none reported on MDR tuberculosis in Rivers State, Nigeria. Therefore, this study set out to assess the proportion of MDR tuberculosis in patients attending some directly observed treatment short-course centres in Port Harcourt, Rivers State, Nigeria.

## Methods

### Ethical considerations

This study was approved by the Rivers State Ministry of Health, Port Harcourt, Rivers State, Nigeria (study approval number: MH/PRS/391/VOL.2/385). Informed verbal consent was obtained from all participants and names were omitted to protect participants’ privacy.

### Study population

Participants between the ages of 18 and 75 years attending six randomly selected directly observed treatment short-course clinics and centres in Port Harcourt, Rivers State, Nigeria, were enrolled in this study from October 2015 to October 2016. These participants comprised people with suspected or presumptive tuberculosis and those recently diagnosed with less than one month duration of tuberculosis therapy. Known tuberculosis patients on therapy for more than one month were excluded from this study.

### Sample collection and processing

Three sputum samples of 5 mL volume were obtained from each participant. These samples were screened for the presence of *M. tuberculosis* using standard microscopic, culture and phenotypic biochemical techniques.

Preliminary detection for acid fast bacilli was carried out by direct microscopic examination following Ziehl-Neelsen staining using a standard protocol. Next, sputum samples were processed for culture using the Petroff’s method^8^ and culture was carried out on Lowenstein-Jensen agar slants. The set-up was incubated at 37 °C and inspected for characteristic growth weekly, for up to six weeks. Phenotypic biochemical identification was then carried out based on catalase enzyme production, nitrate reductase and niacin production.

### Drug susceptibility testing

Drug susceptibility testing was carried out using the Lowenstein-Jensen proportion method.^9,10^ This involved the use of critical drug concentrations of 0.2 *µ*g/mL for isoniazid, 40 *µ*g/mL for rifampicin, 2 *µ*g/mL for ethambutol and 4 *µ*g/mL for streptomycin. Following a 28-day incubation, isolates were documented as resistant if a growth rate exceeding 1% of the control was observed. Multi-drug resistance was defined as resistance to isoniazid and rifampicin.

## Results

A total of 609 participants were enrolled in this study, the majority of which were male (399, 65.5%). Results of the direct microscopy showed that 26 of the participants were smear-positive for acid fast bacilli. However, based on culture and biochemical testing, 30 (4.9%) participants were confirmed as *M. tuberculosis*-positive cases. The rate of tuberculosis was slightly higher among women than among men (Table 1).

**TABLE 1:** Gender-based distribution of culture positive *Mycobacterium tuberculosis*.

Sex	Number	*Mycobacterium tuberculosis*culture results			
		Positive		Negative	
		*n*	%	*n*	%
Female	210	11	5.2	199	94.8
Male	399	19	4.8	380	95.2

**Total**	**609**	**30**	**4.9**	**579**	**95.1**

**FIGURE 1:**
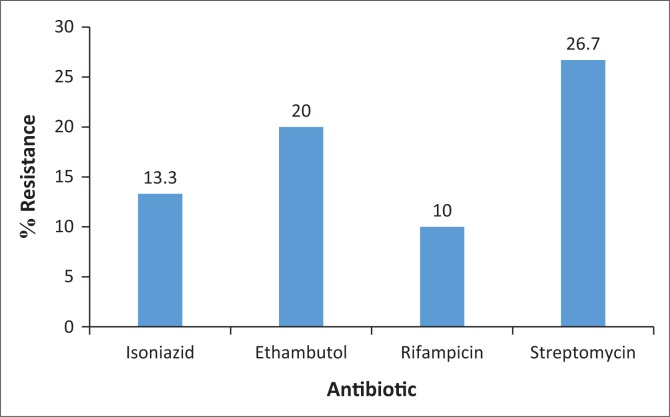
Rates of resistance to first-line tuberculosis drugs.

In this study, 14 isolates (46.7%) were found to be resistant to at least one of the first-line drugs (isoniazid, rifampicin, ethambutol and streptomycin). The highest level of resistance (26.7%) was noted against streptomycin, while the lowest level (10%) was noted against rifampicin (Figure 1).

In total, 11 isolates (36.7%) were mono-resistant and two isolates were resistant to three of the tuberculosis drugs tested (Table 2). MDR tuberculosis was only detected in one case (3.3%). In total, seven different susceptibility profiles were identified in this study.

**TABLE 2:** Drug resistance patterns of *Mycobacterium tuberculosis* isolates.

Resistance profile	Number of isolates	
	*n*	%
**Monoresistant**		
EMB	3	10.0
INH	2	6.7
RIF	1	3.3
STR	5	16.7
**Polyresistant**		
EMB-INH-STR	1	3.3
EMB-RIF-STR	1	3.3
**Multidrug resistant**		
EMB-INH-RIF-STR	1	3.3

EMB, ethambutol; INH, isoniazid; RIF, rifampicin; STR, streptomycin.

## Discussion

With the high tuberculosis burden associated with Nigeria, it could be assumed that a higher burden of MDR tuberculosis would also be reported. However, at present, data on MDR tuberculosis in Nigeria are sparse. Monitoring of this situation is essential for developing and analysing control strategies. Varying rates of MDR tuberculosis have been reported worldwide. These have ranged from very low rates of 0.2% reported from Japan,^11^ to MDR tuberculosis rates as high as 69% reported from Pakistan.^12^ In China, which at 2017 was noted as having the second highest MDR tuberculosis burden,^13^ a study that same year reported a 10.1% total MDR tuberculosis prevalence.^14^ In addition to country to country variations, these rates could vary even within regions. The national Indian MDR tuberculosis rate at 2010 was 2.1%, but a 2013 study analysing East Delhi reported a 1.3% prevalence rate,^15^ while a 15.1% rate was reported among a tribe with a known high tuberculosis prevalence.^16^ Other reported MDR tuberculosis rates from South East Asia include 15.6% from Nepal.^17^ African countries are not among the top three countries associated with over half the global burden of MDR tuberculosis.^18^ The MDR tuberculosis rates from this region have varied, with a range of rates reported: 4.5% from Chad,^19^ 12.0% from Benin,^20^ 5.1% from Mozambique,^21^ 18% from Cameroon,^22^ 11.5% from Djibouti,^23^ and 1.2% and 3.37% from Ethiopia,^24,25^ which are generally higher than the rate observed in this study. In addition to country and regional variations, the rate of MDR tuberculosis is also related to exposure of patients to tuberculosis drugs. Higher rates of MDR tuberculosis are generally reported in retreatment cases as opposed to newly diagnosed cases.^14,17,25,26^

Findings of this current study revealed a 3.3% MDR tuberculosis rate. This figure is lower but similar to the current reported Nigerian MDR tuberculosis WHO data, which estimates a 4.3% rate among new cases.^7,27^ This low rate is encouraging, as a high level of prevalence would constitute a major public health issue. Some previous studies assessing rates of MDR tuberculosis in Nigeria had reported higher rates of 6.9%, 8%, 10.4% and 10.6% from Awka, three different cities, Cross Rivers and Kano respectively and extremely higher rates of 53.6%.^28,29,30,31,32^ Others reported fairly similar rates of 4% from Calabar and Abuja, and 5.2% from Cross Rivers.^33,34,35^ With respect specifically to MDR tuberculosis rates in new cases, results of this study were quite similar to previous reported rates of 3%,^32^ 4%^33^ and 5.2%,^35^ but lower than the pooled rate of 6.0% reported in a recent review on drug resistant tuberculosis in Nigeria.^7^

Despite the relatively low rate of MDR tuberculosis noted in this study, a 46.7% resistance rate against any of the first-line drugs was noted. This value was higher than a 2018 Ethiopian report of a 23.3% resistance rate against any of the first-line drugs.^24^ It was, however, similar to reports from nearby Benin of a 40% rate,^20^ as well as rates of 32.3% and 42% reported from Nigeria.^30,33^ Such a level of drug resistant tuberculosis in this region could complicate patient management by reducing treatment options.

Generally, the lowest rates of resistance have been reported against rifampicin,^19,25,33^ and this was also the finding in this study, with 10% of isolates resistant to rifampicin. The rates of resistance noted against the various drugs in this study (ethambutol, 20%; isoniazid, 13.3%; rifampicin, 10%; streptomycin, 26.7%) are similar to previous reports from Nigeria. The highest level of resistance (26.7%) was noted against streptomycin. This trend has been reported by several other studies assessing drug resistance in tuberculosis cases.^17,22,25,35^ In the study by Otu and colleagues, 47.6% resistance was noted against streptomycin,^33^ while Pokam and colleagues^35^ noted a 27.6% resistance rate against streptomycin. This is thought to be linked with the fact that streptomycin is the oldest tuberculosis drug in use.^36^ While the use of streptomycin in clinical practice is supposed to be limited, this antibiotic is used both as a growth promoter and in therapy in both plant and animal farming.^37^ The development of resistance to this drug in bacteria of clinical importance in non-clinical settings following such use has been described,^38^ and such resistance has further been reported in bacteria of human origin.^39,40^ These may then have the potential to serve as a reservoir for further spread in the clinical setting.

Despite the high resistance to streptomycin, some studies from Cameroon, Ethiopia, and Nepal have reported lower resistance rates of 6.0%, 7.87%, 24.4%, respectively, to streptomycin.^17,22,25^ One of these studies postulated that the lower rate of resistance against streptomycin could be linked with the fact that this drug is no longer a first-line drug for the treatment of tuberculosis in the test region.^25^

### Conclusion

Results of this study show a high level of drug resistance (46.7%) and low level of MDR (3.3%) tuberculosis. The high level of drug resistance is particularly worrisome considering that the test population comprised new cases. This study additionally contributes to the sparse data on drug-resistant tuberculosis in Nigeria, which is necessary for the development of control measures to curb the spread of drug resistant tuberculosis.
